# Draft Genome Sequence of *Lactobacillus fermentum* BFE 6620, a Potential Starter Culture for African Vegetable Foods, Isolated from Fermented Cassava

**DOI:** 10.1128/genomeA.00801-17

**Published:** 2017-08-17

**Authors:** Eliud N. Wafula, Erik Brinks, Biserka Becker, Melanie Huch, Bernhard Trierweiler, Julius M. Mathara, Folarin A. Oguntoyinbo, Gyu-Sung Cho, Charles M. A. P. Franz

**Affiliations:** a Department of Safety and Quality of Fruit and Vegetables, Federal Research Institute of Nutrition and Food, Kiel, Germany; b Department of Microbiology and Biotechnology of the Max Rubner-Institut, Federal Research Institute of Nutrition and Food, Kiel, Germany; c Jomo Kenyatta University of Agriculture and Technology, Food Science and Technology, Nairobi, Kenya; d Department of Microbiology, Faculty of Science, University of Lagos, Akoka, Lagos, Nigeria

## Abstract

We report the draft genome sequence of *Lactobacillus fermentum* BFE 6620 from fermented cassava used as a potential starter culture for African vegetable fermentation. Sequence analysis showed the assembled genome size to be 1,982,893 bp, encoding a predicted total of 2,003 protein-coding genes, 14 rRNAs, 54 tRNAs, and 3 noncoding RNAs (ncRNAs).

## GENOME ANNOUNCEMENT

*Lactobacillus fermentum* is a heterofermentative lactic acid bacterium belonging to the *Bacilli* class of the phylum *Firmicutes* and the family *Lactobacillaceae*. This species occurs in diverse habitats, including the human gut, milk products, fermenting plant material, and animals (1). It is considered to be a good probiotic candidate, due to its ability to withstand gastrointestinal conditions (2), and was reported to have potential for prevention of community-acquired infections (3), modulation of the immune system, and production of antimicrobial compounds (4).

*Lactobacillus fermentum* BFE 6620 was isolated from fermented cassava for production of gari in Benin. This strain, together with *Lactobacillus plantarum* BFE 5092, was successfully used as a starter culture in the fermentation of African kale leaves (5). There are currently 25 *L. fermentum* genome sequences reported, of which 6 were completely sequenced. The genome of strain BFE 6620 was sequenced in order to assess its technological and functional properties for vegetable food fermentation and to compare its genome sequence with already sequenced *L. fermentum* strains from different sources.

The total genomic DNA of *L. fermentum* BFE 6620 was isolated using the peqGOLD bacterial DNA kit (Peqlab, Erlangen, Germany). The sequencing library was prepared with an Illumina Nextera XT library prep kit (Illumina, San Diego, CA, USA) and run on the MiSeq with 2 × 251 paired ends. In total, 2,429,489 paired-end sequence reads were obtained with an approximately 242-fold coverage, and the reads were assembled *de novo* using SPAdes version 3.10.1 (6). The draft genome assembly consisted of 149 scaffolds, and the *N*_50_ was 35,982. The genome size of *L. fermentum* BFE 6620 is 1,982,893 bp, with a 52.1 mol% G+C content. The genome sequence was annotated using the Rapid Annotations Subsystems Technology (RAST) and NCBI (7) servers. The sequence contained 2,003 protein-coding sequences, 14 rRNAs, 54 tRNAs, and 3 noncoding RNAs (ncRNAs). No acquired antibiotic resistance genes were found using ResFinder server (v. 2.1) (8). With the use of the RAST server, draft genome comparison with reference strain *L. fermentum* IFO3956 (GenBank accession no. AP008937) showed that the *L. fermentum* BFE 6620 contained 88 coding genes for proteins involved in phosphoenolpyruvate/phosphotransferase (PEP/PTS) systems for utilization of trehalose, a malolactic enzyme, and a pyridoxamine 5′-phosphate oxidase (involved in vitamin B_6_ biosynthesis), which were not present in the reference strain.

### Accession number(s).

This whole-genome shotgun project has been deposited at DDBJ/ENA/GenBank under the accession no. NIWV00000000.
